# Application of thromboelastography to evaluate the effect of different routes administration of tranexamic acid on coagulation function in total hip arthroplasty

**DOI:** 10.1186/s13018-019-1497-y

**Published:** 2019-12-11

**Authors:** Xingming Xu, Jiang Jiang, Wei Liu, Xiaofeng Li, Huading Lu

**Affiliations:** 1 Department of Orthopaedic, The Fifth Affiliated Hospital Of Sun Yat-Sen University, No. 52, Meihua East Road, Zhuhai, 519000 Guangdong China; 2 Department of Orthopaedics, The First Affiliated Hospital Of Nanchang University, No. 17, Yongwaizheng Street, Nanchang, 330006 Jiangxi China

**Keywords:** Tranexamic acid, Total hip arthroplasty, Thromboelastography, Coagulation

## Abstract

**Background:**

Tranexamic acid (TXA) is widely used to reduce blood loss and transfusion rates in total hip arthroplasty(THA). Thromboelastography, which can monitor coagulation changes from clotting to fibrinolysis dynamically. In this study, thromboelastography was used to assess the dynamic changes in the coagulation of patients who underwent THA with the administration of TXA.

**Methods:**

This randomized controlled trial consisted of 207 consecutive patients who underwent primary total hip arthroplasty. Patients were randomized into three groups: topical-TXA group received a topical application of TXA, IV-TXA group received an intravenous injection of TXA, and control group. Thromboelastography was performed 1 day before surgery and first, fourth, seventh days after surgery. The primary outcomes were thromboelastography parameters, the rates of deep vein thrombosis(DVT), and pulmonary embolism(PE). Secondary outcomes included perioperative blood loss, transfusion rates, and other perioperative complications.

**Results:**

The mean calculated total blood loss in the Topical-TXA group were 832.7 ± 279.84 ml and 834.8 ± 322.94 ml in the IV-TXA group, which were significantly reduced (*p* < 0.05) compared with control groups at 1093.3 ± 379.7 ml. There were no significant differences between topical-TXA and IV-TXA groups in total blood loss or transfusion rates. *K* and *R* have reached a nadir from preoperative levels to 4th day postoperatively and then began to increase.α angle and CI peaked from preoperative levels to the fourth day postoperatively and then began to decline.IV-TXA significantly (*p* < 0.05) promoted coagulation levels compared with topical-TXA and control groups in the early postoperative period. Almost no significant differences were observed between topical-TXA and control groups in thromboelastography parameters.No significant differences were observed in the incidence of thromboembolic complications and other perioperative complications.

**Conclusions:**

The topical administration of TXA had the same hemostatic effect as intravenous injection tranexamic acid. Coagulation function peaked on 4th day postoperatively and then began to decline. IV-TXA was more enhanced coagulation functions compared with topical-TXA.

## Background

Total hip arthroplasty (THA) is currently recognized as an effective treatment for patients with hip joint disease. However, THA has been associated with considerable blood loss and massive transfusion requirements. Large number methods of controlling bleeding such as thromboplastic agents, topical freezing saline, deliberate hypotension and administration of fibrinolytic inhibitors as aprotinin and tranexamic acid (TXA) have been widely used [1–3].

Tranexamic acid (TXA) is an antifibrinolytic drug, which is widely used in total hip arthroplasty(THA) [4–6]. Many studies and meta-analyses have demonstrated that the administration of TXA could effectively decrease blood loss and transfusion rate in THA [7–10]. However, one of the major concerns for TXA is the potential increased risk of thrombosis, and that possibility cannot be overlooked. The ideal method of delivery and dosage remains controversial.

Although, ultrasound examinations are routinely performed from a macroscopic perspective and conventional coagulation assays are performed in patients who undergo THA have confirmed the safety of TXA, the dynamic changes in coagulation, platelets function, and fibrinolysis in patients administered tranexamic acid are not considered. Thromboelastography (TEG), as a blood clotting test, which can monitor coagulation changes from clotting to fibrinolysis dynamically, was widely used in the field of transplantation and blood vasculature operations [11–13]. TEG can simulate the whole process of coagulation and fibrinolysis in the human body environment, which is more sensitive than conventional coagulation assays in the evaluation of hypercoagulation, low coagulation, and fibrinolysis [14–16].

Thus, this study aimed to assess the dynamic changes in the coagulation of patients who undergo THA with the administration of TXA, to identify possible complications, and to provide an ideal application of TXA.

## Materials and methods

This prospective randomized study was approved by the local ethics committee. Prior to starting this trial, informed consent was obtained from all of the patients.

### Inclusion and exclusion criteria

Patients were diagnosed with osteoarthritis, and those who underwent unilateral primary cementless THA were prospectively enrolled in this trial between October 2016 and July 2018. The exclusion criteria included (1) if they had a history of hemorrhagic blood diseases; (2) pre-operative anticoagulant treatment within 7 days of the surgery; (3) allergy to TXA, renal, or hepatic impairment; (4) patients with a known of the thrombotic disorder. All the included patients underwent 6 months follow-up postoperatively.

### Grouping and drug delivery

Computerized block randomization was performed by an independent research assistant who was not involved in the study. Opaque sealed envelopes containing a number were opened after the patients arrived in the operating room. The numbers in the envelopes were created using a computer-generated randomization list. One of our researchers, who was not involved in the operation, was responsible for opening the envelopes and preparing the appropriate medication. Then, patients were allocated into three groups: (1) 2 g TXA in 50 ml normal saline topical injection of TXA into articular cavity of hip after the uncemented prosthesis was installed and joint capsule closed (topical-TXA group); (2) 20 mg/kg TXA in 100 ml normal saline delivered by intravenous injection 5 min prior to skin incision (IV-TXA group); (3) and the control group. For all the groups, the same amounts of saline solution were applied at the same time points (if not IV injection or topical administration of TXA).

All the surgeries were performed under continuous lumbar epidural anesthesia. All surgeries were performed by the same surgeon and all the operations were completed using the posterolateral hip approach. The prosthesis was a cementless acetabular cup (Trilogy IT Acetabular System, Shell with Cluster Holes Porous, Zimmer, USA) and a cementless femoral stem (VerSys Hip System, Fiber Metal Taper, Zimmer, USA). A drain was placed in all groups and clamped for 2 h. The drainage was removed 48 h after the operation. All the patients, surgeons, and outcome assessors were blinded to the randomization. All patients started passive and active function training after anesthesia resolution. An inflatable lower-extremity venous pump was applied on the first day after surgery until hospital discharge. Ten milligrams of rivaroxaban was given orally once daily for 35 days for venous thromboembolism prophylaxis.

### Blood transfusion protocol

Patients received blood transfusions based on the following criteria: (1)patients with Hb levels less than 70 g/l; (2)patients with Hb levels more than 70 g/l but with a poor mental state or other signs of distress such as pallor, polypnea, hypotension, palpitation, or dizziness.

### Outcome measures

Demographic data were collected before the operation including age, gender, height, and weight. Body mass index(BMI) is the weight(kg) divided by the square of height (m^2^). Hematocrit (Hct), fibrinogen (FIB), prothrombin time (PT), and activated partial thromboplastin time (APPT) were measured before and after the operation. The primary outcomes were TEG parameters, the rates of deep vein thrombosis(DVT), and pulmonary embolism (PE). Blood samples of 2 ml were extracted from all patients who met the criteria before the operation and first, fourth, and seventh days after the operation, respectively. Blood was then examined by TEG (CFMS LEPU-8800, LEPU TECHNOLOGY, China), which produced five parameters including reaction time (*R* value), coagulation time (*K* value), alpha angle (α angle), maximum amplitude (MA), and coagulation index (CI). *R* is the time elapsed until the first evidence of a clot. *K* is the time from the end of *R* until the clot reaches 20 mm and represents the speed of clot formation. α angle is the angle of the curve made as *K* is reached and offers information like that from *K*. The maximum amplitude (MA)is an indication of the maximum strength of the clot. A mathematical formula can be used to determine a coagulation index (CI) (or overall assessment of coagulability) that takes of these parameters into account [17].In TEG, hypercoagulability can be defined as CI > + 3. All patients underwent a routine ultrasound examination to screen for DVT on the fourth and seventh days after the operation. If the patients were suspected of pulmonary embolism(PE) based on clinic signs such as acute dyspnea or unexplained hypoxemia,a CT pulmonary angiography was performed to confirm the diagnosis. Signs of symptomatic DVT and pulmonary embolism (PE) were continuously monitored for 6 months by follow-up examinations.

The secondary outcomes were total volume of drainage, intraoperative blood loss, total blood loss, and other perioperative complications (including wound infection, wound hematoma, and dislocation). The staff who evaluated the results was blinded to the allocation. The blood volume of each patient was calculated according to the formula of Nadler et al. [18]. Total blood loss was calculated according to the formula of Gross [19].

### Statistical analysis

All quantitative data were checked for homogeneity of variance and the statistical significance between groups was determined by one-way ANOVA. Pearson’s chi-square test or Fisher’s exact test were used to analyze the qualitative variables. The statistical analysis was performed using SPSS software (version 19.0; SPSS). A *p* value < 0.05 was considered significant.

## Results

### Patients

A total of 256 patients were screened for eligibility from October 2016 to July 2018. Thirty-four patients were excluded on the basis of our exclusion criteria, and 15 patients declined to participate. Two hundred seven patients were analyzed at last. Seventy-two patients were randomized to topical-TXA group, 68 patients were randomized to IV-TXA group, and 67 patients were randomized to the control group. The duration of postoperative follow-up in this study was 6 months. The demographic and pre-and postoperative characteristics of the patients are shown in Table 1. There were no statistically significant differences among the groups. This included no differences in baseline age, gender, body mass index, preoperative laboratory indices, or postoperative complications (deep vein thrombosis, pulmonary embolism, wound infection, and wound hematoma or dislocation).

**Table 1:** Characteristics of the patients

	Topical-TXA (*n* = 72)	IV-TXA (*n* = 68)	Control (*n* = 67)	
	Mean ± SD	Mean ± SD	Mean ± SD	*p* value
Demographic characteristics				
Age (years)	61.9 ± 6.1	62.5 ± 6.6	63.3 ± 6.3	0.407
Gender male/female	34/38	32/36	35/32	0.790
Body mass index(kg/m^2^)	22.3 ± 2.7	22.0 ± 2.3	22.2 ± 2.5	0.637
Preoperative laboratory index				
Prothrombin time (s)	10.6 ± 0.9	10.6 ± 0.7	10.7 ± 0.73	0.889
APTT (s)	28.8 ± 4.2	29.1 ± 3.9	29.3 ± 3.5	0.735
Plasma fibrinogen (g/L)	2.9 ± 0.5	2.9 ± 0.6	2.9 ± 0.5	0.649
Postoperative complications				
Number of DVT	1	0	0	0.346
Number of PE	0	1	0	0.327
Wound infection	0	0	0	1.000
Wound hematoma	0	1	1	0.423
Dislocation	0	0	0	1.000

*APTT* activated partial thromboplastin time, *DVT* deep vein thrombosis, *PE* pulmonary embolism

### Blood loss and transfusions

**Fig. 1:**
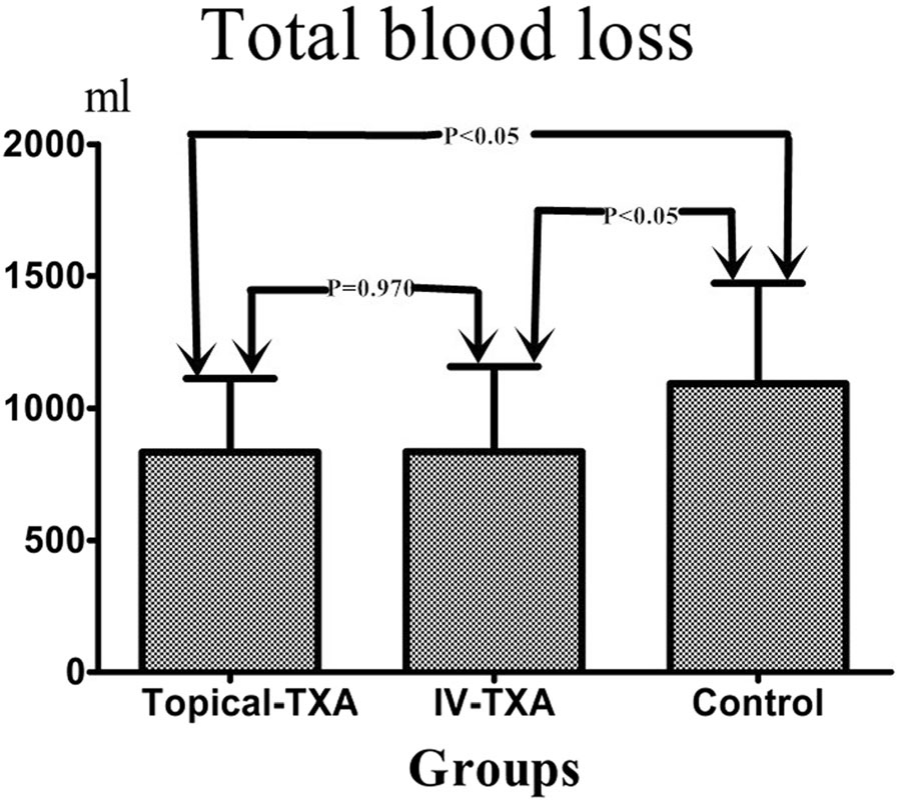
The histogram demonstrated that the mean total blood loss in topical-TXA or IV-TXA group was significantly less than control groups(*P*<0.05).There was not significant difference between IV-TXA and topical-TXA groups(*P* value =0.970)

**Fig. 2:**
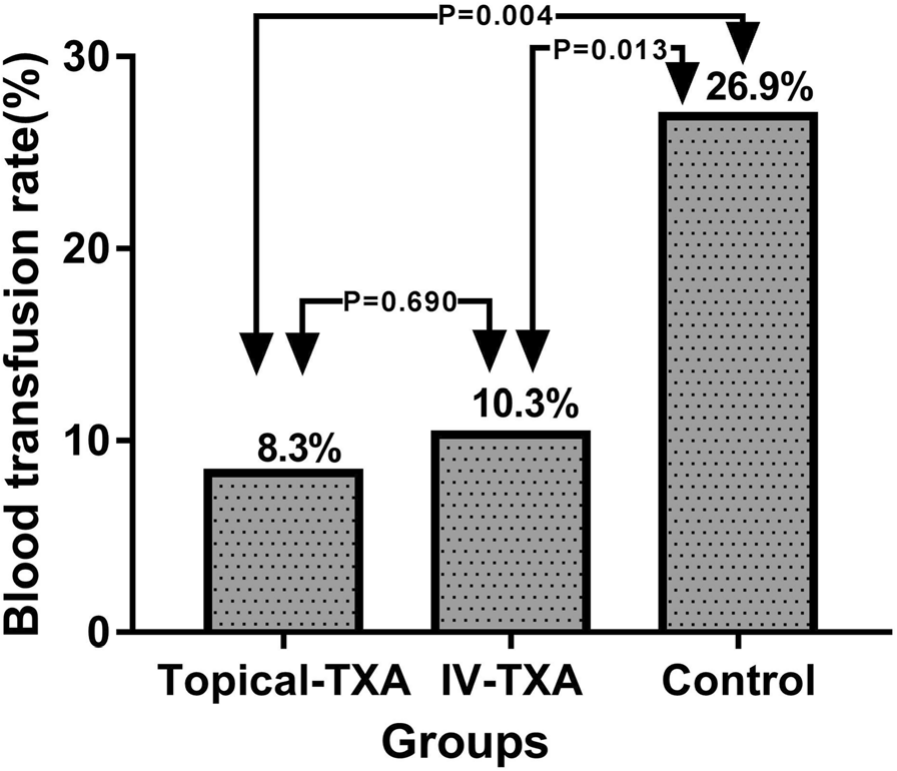
The histogram demonstrated that the transfusion rates in topical-TXA or IV-TXA group was significantly less than control groups(P=0.004 and P=0.013).There was not significant difference between IV-TXA and topical-TXA groups(*P*=0.690)

The mean total blood loss in the topical-TXA, IV-TXA, and control groups were 832.7 ± 279.8, 834.8 ± 322.9, and 1093.3 ± 379.7 ml, respectively (Table 2 and Fig. 1). The topical-TXA group and IV-TXA group had significantly lower amounts of total blood loss than the control group (*p* < 0.05). There were no significant differences between topical-TXA and IV-TXA groups in total blood loss (*p* value = 0.970). The postoperative total volume of drainage was significantly lower in the topical-TXA group than in the other two groups (*p* value < 0.05 for both) (Table 2). The mean amount of intraoperative blood loss was 166.7 ± 42.5 ml in the topical-TXA group, 168.0 ± 47.4 ml in the IV-TXA group and 176.7 ± 46.2 ml in the control group, with no significant intergroup differences. Six patients in the topical-TXA group, 7 patients in the IV-TXA group, and 18 patients in the control group required transfusions. These differences were statistically significant (*p* value = 0.004) (Table 2 and Fig. 2).

**Table 2:** The major outcomes of three groups

	Topical-TXA (*n* = 72)	IV-TXA (*n* = 68)	Control (*n* = 67)	
	Mean ± SD	Mean ± SD	Mean ± SD	P-value
Intraoperative blood loss (ml)	166.7 ± 42.5	168.0 ± 47.4	176.7 ± 46.2	0.379
Total blood loss (ml)	832.7 ± 279.8*	834.8 ± 322.9#	1093.3 ± 379.7	<0.001
Total volume of drainage (ml)	91.5 ± 48.0$*	294.4 ± 149.7	313.3 ± 141.5	<0.001
Number of blood transfusion	6(8.3%) *	7(10.3%)#	18(26.9%)	0.004

^$^Significant difference (*p* < 0.05) between topical-TXA and IV-TXA groups

*Significant difference (*p* < 0.05) between topical-TXA and control groups

^#^Significant difference (*p* < 0.05) between IV-TXA and control groups

### Thromboelastograph parameters

**Fig. 3:**
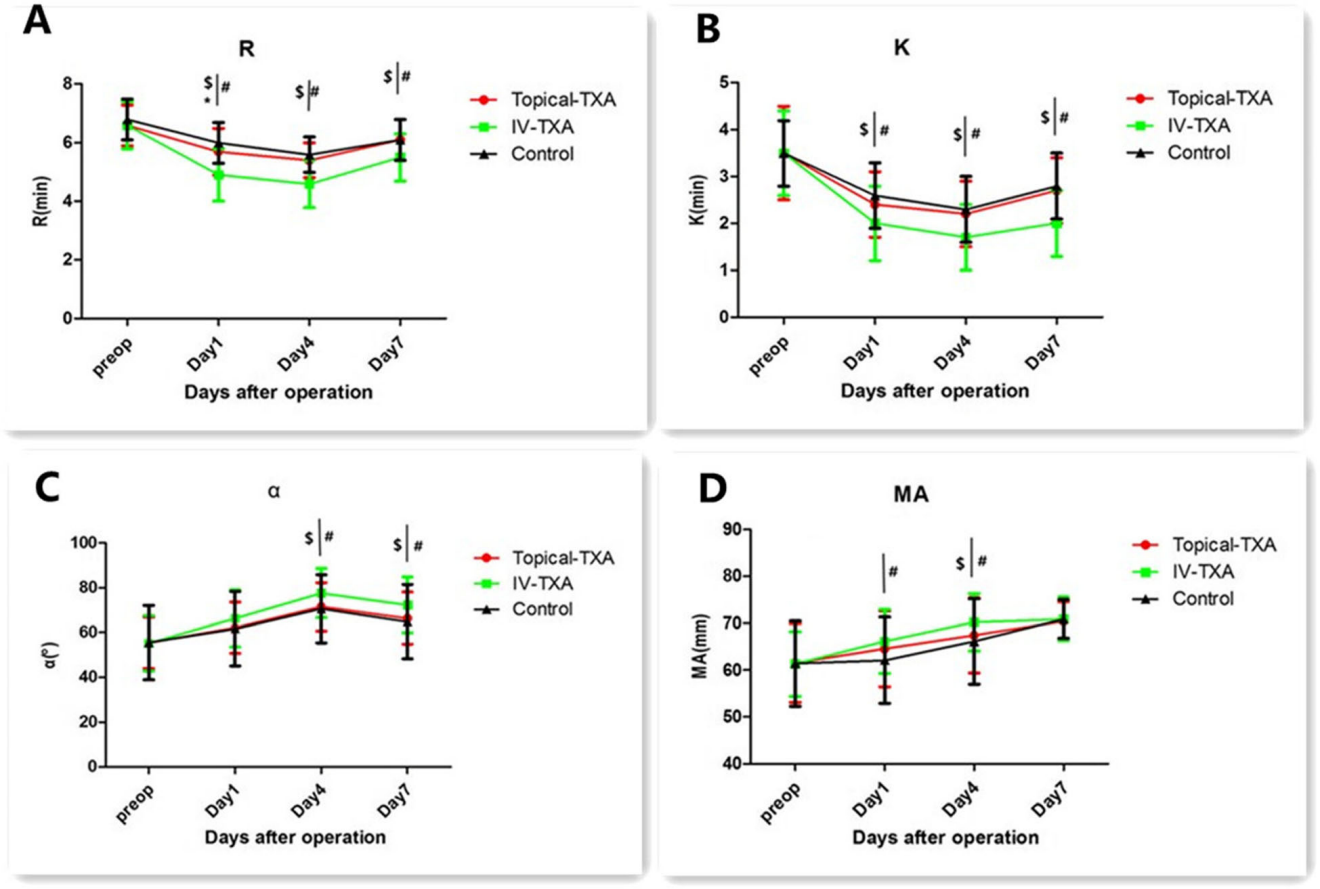
Perioperative thromboelastographic parameters analysis:R time(**a**), K time (**b**), α angle(**c**), and maximum amplitude(MA) value (**d**). The error bars indicate standard deviation.$Significant difference (*p* < 0.05) between Topical-TXA and IV-TXA groups. *Significant difference (*p* <0.05) between Topical-TXA and Control groups. #Significant difference (*p* < 0.05) between IV-TXA and Control groups.Preop=preoperative

**Fig. 4:**
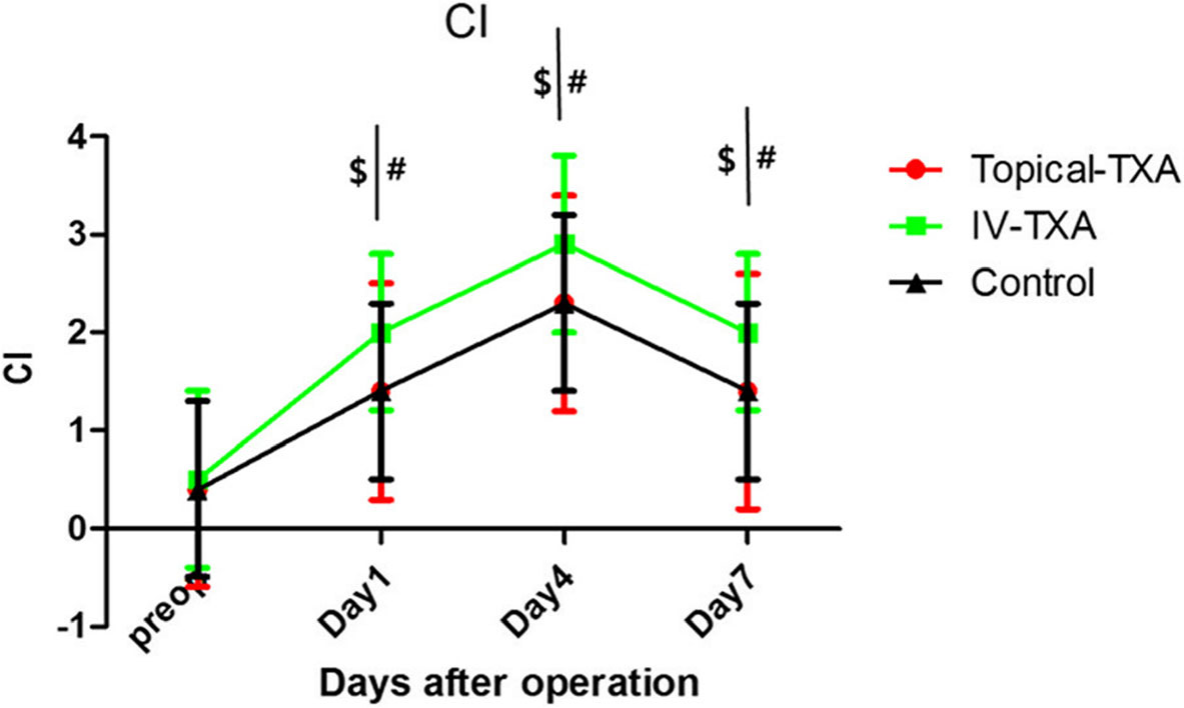
Perioperative thromboelastographic CI analysis.The error bars indicate standard deviation.$Significant difference (p < 0.05) between Topical-TXA and IV-TXA groups.*Significant difference (p <0.05) between Topical-TXA and Control groups.#Significant difference (p < 0.05) between IV-TXA and Control groups.Preop=preoperative.CI=Coagulation index

There were no significant differences in *R*, *K*, α angle, MA, or CI among groups preoperatively (Table 3,Fig. 3, and Fig. 4). *K* and *R* have reached a nadir from preoperative levels to the fourth day postoperatively and then began to increase.

**Table 3:** Thromboelastography on preoperatively , postoperative day 1, 4, and 7

	Topical-TXA(*n* = 72)	IV-TXA (*n* = 68)	Control(*n* = 67)	
Items	Mean ± SD	Mean ± SD	Mean ± SD	*p* value
*R* (min)				
Preoperatively	6.6 ± 0.7	6.6 ± 0.8	6.8 ± 0.7	0.247
Postoperative				
Day 1	5.7 ± 0.8$*	4.9 ± 0.9#	6.0 ± 0.7	0.000
Day 4	5.4 ± 0.6$	4.6 ± 0.8#	5.6 ± 0.6	0.000
Day 7	6.1 ± 0.7$	5.5 ± 0.8#	6.1 ± 0.7	0.000
*K* (min)				
Preoperatively	3.5 ± 1.0	3.5 ± 0.9	3.5 ± 0.7	0.904
Postoperative				
Day 1	2.4 ± 0.7$	2.0 ± 0.8#	2.6 ± 0.7	0.000
Day 4	2.2 ± 0.7$	1.7 ± 0.7#	2.3 ± 0.7	0.000
Day 7	2.7 ± 0.7$	2.0 ± 0.7#	2.8 ± 0.7	0.000
MA (mm)				
Preoperatively	61.5 ± 8.4	61.3 ± 6.9	61.4 ± 9.1	0.989
Postoperative				
Day 1	64.5 ± 8.1	66.1 ± 6.8#	62.1 ± 9.2	0.017
Day 4	67.4± ± 8.0$	70.2 ± 6.1#	66.1 ± 9.1	0.010
Day 7	70.4 ± 4.1	70.9 ± 4.6	70.9 ± 4.1	0.695
α (°)				
Preoperatively	55.4 ± 11.4	55.2 ± 12.3	55.5 ± 16.5	0.988
Postoperative				
Day 1	62.1 ± 11.4	66.3 ± 12.7	61.7 ± 16.6	0.092
Day 4	71.5 ± 10.8$	77.6 ± 10.9#	70.6 ± 15.2	0.002
Day 7	66.4 ± 11.6$	72.4 ± 12.5#	64.9 ± 16.6	0.004
CI				
Preoperatively	0.4 ± 1.0	0.5 ± 0.9	0.4 ± 0.9	0.803
Postoperative				
Day 1	1.4 ± 1.1$	2.0 ± 0.8#	1.4 ± 0.9	0.000
Day 4	2.3 ± 1.1$	2.9 ± 0.9#	2.3 ± 0.9	0.001
Day 7	1.4 ± 1.2$	2.0 ± 0.8#	1.4 ± 0.9	0.000

^$^Significant difference (*p* < 0.05) between topical-TXA and IV-TXA groups

*Significant difference (*p* < 0.05) between topical-TXA and control groups

^#^Significant difference (*p* < 0.05) between IV-TXA and control groups

α angle and CI peaked from preoperative levels to the fourth day postoperatively and then began to decline. IV-TXA group enhanced coagulation peak values (*P* < 0.05), but it did not obviously change the coagulation tendency in general. MA increased steadily from preoperative levels to the seventh day postoperatively.IV-TXA significantly (*p* < 0.05) promoted coagulation levels in the early postoperative period (Table 3, Fig. 3, and Fig. 4). Almost no significant differences were observed between topical-TXA and control groups (except for the *R* on the first day postoperatively).

## Discussion

Tranexamic acid has been widely used in artificial hip replacement [4–10, 20], but there is still controversy over the method of delivery and the optimal dosage of TXA administration. The advantage of topical TXA was considered to be less systemic absorption, ease of administration, and better concentration of TXA at the bleeding site [21–23]. However, some studies have reported that topical administration of TXA can reduce blood loss and the transfusion rate but not achieve ideal satisfaction, and they hypothesized that intravenous (IV) infusion of TXA is more predictable than the topical application for reaching maximum efficacy [22, 24]. In the present study, we demonstrated that intravenous injection of tranexamic acid and topical application of tranexamic acid have the same hemostatic effect in patients undergoing primary THA, which supports a recent meta-analysis [25]. The routine dose of topical tranexamic acid is 1 g to 3 g and intravenous tranexamic acid is 10 mg/kg to 30 mg/kg in THA [3, 25, 26]. In this study, we selected a dose of 2 g for topical application in the topical-TXA group and 20 mg/kg for intravenous injection in the IV-TXA group.

In this study, there was one patient who developed deep vein thrombosis in the topical-TXA group, one developed pulmonary embolism in the IV-TXA group, and no patient developed a thromboembolic complication in control group. Although, there were no statistically significant differences among the groups in thromboembolic complications, the effect of tranexamic acid on the coagulation system is still unknown.

Traditional coagulation indices, including prothrombin time (PT), activated partial thromboplastin time(APTT), fibrinogen (FIB), and international normalized ratio (INR), which were widely used to monitor coagulation status. However, these traditional indices are mainly representing the initial functions of coagulation factors, which were also time-consuming. Thromboelastography [27], as a coagulation-monitoring method, which was widely used in the field of transplantation and blood vasculature operations [13, 28], monitors the dynamic process of coagulation and fibrinolysis. Zavlin et al. [14] concluded that TEG is more dynamic compared to the APTT or PT, and is exceedingly reflective of real-time coagulation status changes. While these traditional coagulation tests were not able to identify patients with thrombosis in the fields of microsurgery. Hans et al [29] also showed that TEG provides insight into the speed and strength of clot formation as well as fibrinolysis.

In this study, TEG was used to dynamically monitor coagulation from preoperation to post-operation seventh day. Thromboelastographic analysis demonstrated that coagulation function peaked on the fourth day postoperatively and then began to decline among groups. Thromboelastogram showed that intravenous injection TXA promotes hypercoagulable state (significant decrease in *R*, *K*, and significant increase in α angle, CI), which was similar to what Emara et al. reported [30]. IV-TXA increased the coagulation peak values, but it did not show an obvious change in the coagulation tendency. Maximum amplitude analysis demonstrated that stronger platelet aggregation function among groups from preoperative levels to 7th day postoperation. The present study observed nearly no significant differences between topical-TXA and control groups (except for the *R* on the first day of postoperation). That is because the topical application of tranexamic acid is less systemic absorption [31], and that has little effect on the coagulation system.

Recently, Wu et al. [32] retrospectively reviewed 359 primary total joint arthroplasty patients to evaluate the coagulation function. They concluded that multiple-dose of TXA was associated with the aggravated hypercoagulable state when compared with a single dose of TXA. Although this prothrombotic state did not provoke thrombosis, prolonged high systemic TXA levels associated with multiple injections or continuous infusion regimens may induce venous thromboembolic (VTE) events and that must be taken into consideration. In the present study, only a single dose intravenous injection with a single topical application of TXA was compared and concluded that IV-TXA was more enhanced coagulation. A prospective randomized controlled study with a large scale of patients is required in multiple-dose of the TXA field.

There were some limitations in the present study. First, the number of patients was small. Larger sample size is needed to analyze the effect of tranexamic acid on TEG parameters and to detect possible complications. Second, the postoperative TEG evaluation was performed only From the first day after surgery to the seventh day after surgery, a long postoperative dynamic monitoring is needed. Third, we do not know exactly how much TXA has permeated through articular tissue surface to the systemic circulation since serum concentrations of TXA were not measured. Fourth, asymptomatic deep vein thrombosis and pulmonary embolism were not routinely given ultrasound examination or performed routine screening during 6 months follow-up. However, we did continuously observed thromboembolic complications based on their clinical symptoms. Last, we cannot be certain whether TXA has a negative effect on the osseous integration of the implants or on joint wear.

In conclusion, the present study demonstrated that the topical administration of TXA had the same hemostatic effect as intravenous tranexamic acid on reducing perioperative total blood loss. Coagulation function peaked on the fourtth day postoperatively and then began to decline. IV-TXA was more enhanced in coagulation functions compared with topical-TXA, but it did not change the coagulation tendency.TEG is a reliable tool to understand the coagulation status of patients undergoing THA.

## Data Availability

The datasets used and/or analyzed during the current study are available from the corresponding author on reasonable request.

## Abbreviations

APPT: Activated partial thromboplastin time
CI: Coagulation index
DVT: Deep vein thrombosis
FIB: Fibrinogen
Hct: Hematocrit
INR: International normalized ratio
MA: Maximum amplitude
PE: Pulmonary embolism
PT: Prothrombin time
R: Reaction time
TEG: Thromboelastography
THA: Total hip arthroplasty
TXA: Tranexamic acid
